# Supplementary material for: Identifying COVID-19 peaks using early warning signals
Source: PLoS Comput Biol. 2025 Sep 24;21(9):e1013524. doi: 10.1371/journal.pcbi.1013524 (PMC12483279; doi:10.1371/journal.pcbi.1013524)
Supplement: S1 Text — (PDF) [file pcbi.1013524.s001.pdf]

SIS Model Equations

The compartment diagram for the SIS model is given in Fig. S1 below. Previous analyses have considered an immigration force of infection (η), but we instead considered the effective transmission rate (β) between susceptible (S) and infectious (I) individuals as the control parameter [1–3].

Assuming a constant population size of N (such that there is no demography/births and deaths), this model, which has $R_0 = \beta/\gamma$ (where γ is the recovery rate), has two fixed points: a disease-free equilibrium ($S^* = N$); and an endemic equilibrium $\left(S^* = N/R_0 = N\gamma/\beta, I^* = N(1 - 1/R_0)\right)$. The stability of each is also given by R_0, with the endemic equilibrium (disease-free equilibrium) being stable (unstable) for $R_0 > 1$ and unstable (stable) for $R_0 < 1$.

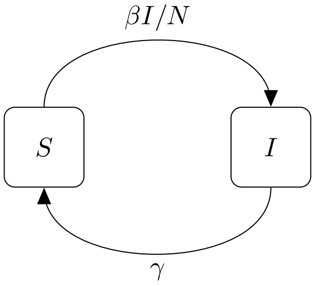

Fig S1. SIS compartmental diagram showing the infection and recovery processes. Transmission occurs at a rate of β, and recovery occurs at a rate of γ.

The corresponding system is then one-dimensional (assuming a constant population size such that $S + I = N$, where N is the total population size) with the ordinary differential equation (ODE) for I as:

$$\frac{\mathrm{d}I}{\mathrm{d}t} = \beta(N - I)I/N - \gamma I. \tag{S1}$$

We use the system size expansion and linear noise approximation to express the relationship:

$$I = N\phi(t) + N^{1/2}\zeta, \tag{S2}$$

between I, its expected proportion $\phi(t) = \langle I \rangle / N$ and the deviations, $\zeta(t)$, from this proportion (as expected from the law of large numbers [4]).

To first order (in N), the system can then be expressed as:

$$\frac{\mathrm{d}\phi}{\mathrm{d}t} = \beta\phi(1 - \phi) - \gamma\phi. \tag{S3}$$

The Jacobean of this system is found as: $J = \beta(1 - 2\phi) - \gamma$ and the noise-covariance matrix is given by $B = \beta\phi(1 - \phi) - \gamma\phi$. Thus, we can find the FPE and corresponding SDE as:

$$\frac{\partial \Pi(\zeta, t)}{\partial t} = (\beta(1 - 2\phi) - \gamma)\frac{\partial \zeta \Pi}{\partial \zeta} + \frac{1}{2}(\beta\phi(1 - \phi) - \gamma\phi)\frac{\partial^2 \Pi}{\partial \zeta^2}, \tag{S4}$$

$$d\zeta = (\beta(1 - 2\phi) - \gamma)\zeta dt + \sqrt{\beta\phi(1 - \phi) + \gamma\phi}\, dW_t, \tag{S5}$$

agreeing with previous results [1, 2, 5].

Taking the moments of the FPE, the variance and other statistics can then be calculated.

References

1. Southall E, Brett TS, Tildesley MJ, Dyson L. Early warning signals of infectious disease transitions: a review. *Journal of The Royal Society Interface* **18**(182):20210555 (2021). doi:10.1098/rsif.2021.0555.

2. O'Regan SM, Drake JM. Theory of early warning signals of disease emergence and leading indicators of elimination. *Theoretical Ecology* **6**(3):333–357 (2013). doi:10.1007/s12080-013-0185-5.

3. Brett TS, Drake JM, Rohani P. Anticipating the emergence of infectious diseases. *Journal of The Royal Society Interface* **14**(132):20170115 (2017). doi:10.1098/rsif.2017.0115.

4. Kampen NGv. *Stochastic processes in physics and chemistry.* North-Holland personal library. Elsevier, Amsterdam ; Boston, 3rd ed edition (2007). OCLC: ocm81453662.

5. Gama Dessavre A, Southall E, Tildesley MJ, Dyson L. The problem of detrending when analysing potential indicators of disease elimination. *Journal of Theoretical Biology* **481**:183–193 (2019). doi:10.1016/j.jtbi.2019.04.011.
