# Supplementary material for: Identifying COVID-19 peaks using early warning signals
Source: PLoS Comput Biol. 2025 Sep 24;21(9):e1013524. doi: 10.1371/journal.pcbi.1013524 (PMC12483279; doi:10.1371/journal.pcbi.1013524)
Supplement: S2 Fig — Proportion infectious over time for the ten thousand simulations run for each of the four modelling scenarios (constant β(t), increasing β(t), decreasing β(t) and a step-decrease in β(t)). (a) Alpha-like pathogen. (b) Wild-type-like pathogen. (PDF) [file pcbi.1013524.s002.pdf]

Simulation Trajectories

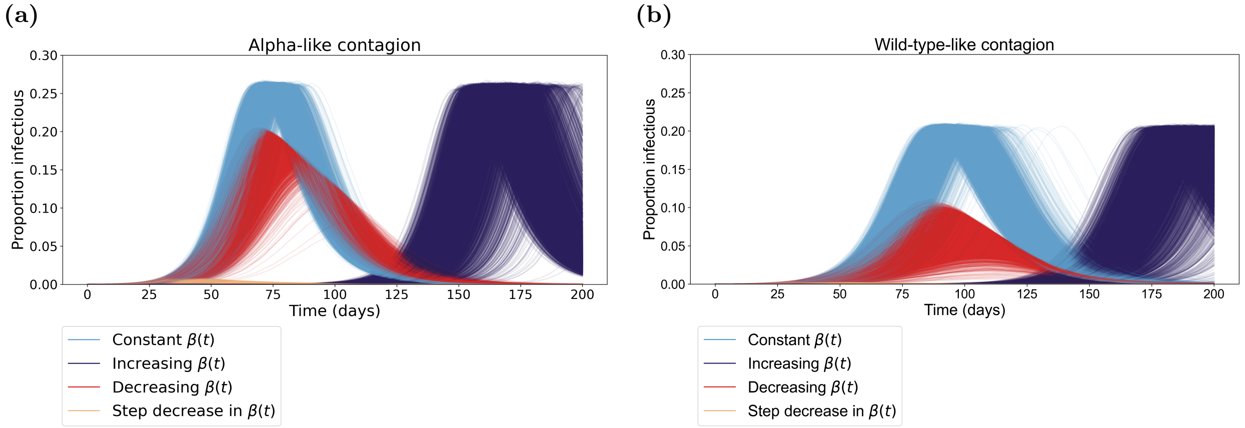

Fig S2A. Proportion infectious over time for the ten thousand simulations run for each of the four modelling scenarios (constant $\beta(t)$, increasing $\beta(t)$, decreasing $\beta(t)$ and a step-decrease in $\beta(t)$). **(a)** Alpha-like pathogen. **(b)** Wild-type-like pathogen.
