# Supplementary material for: Identifying COVID-19 peaks using early warning signals
Source: PLoS Comput Biol. 2025 Sep 24;21(9):e1013524. doi: 10.1371/journal.pcbi.1013524 (PMC12483279; doi:10.1371/journal.pcbi.1013524)
Supplement: S4 Fig — Plots for each of the four modelling scenarios (constant β(t), increasing β(t), decreasing β(t) and a step-decrease in β(t)) at three different time points. (PDF) [file pcbi.1013524.s004.pdf]

Q–Q Plots

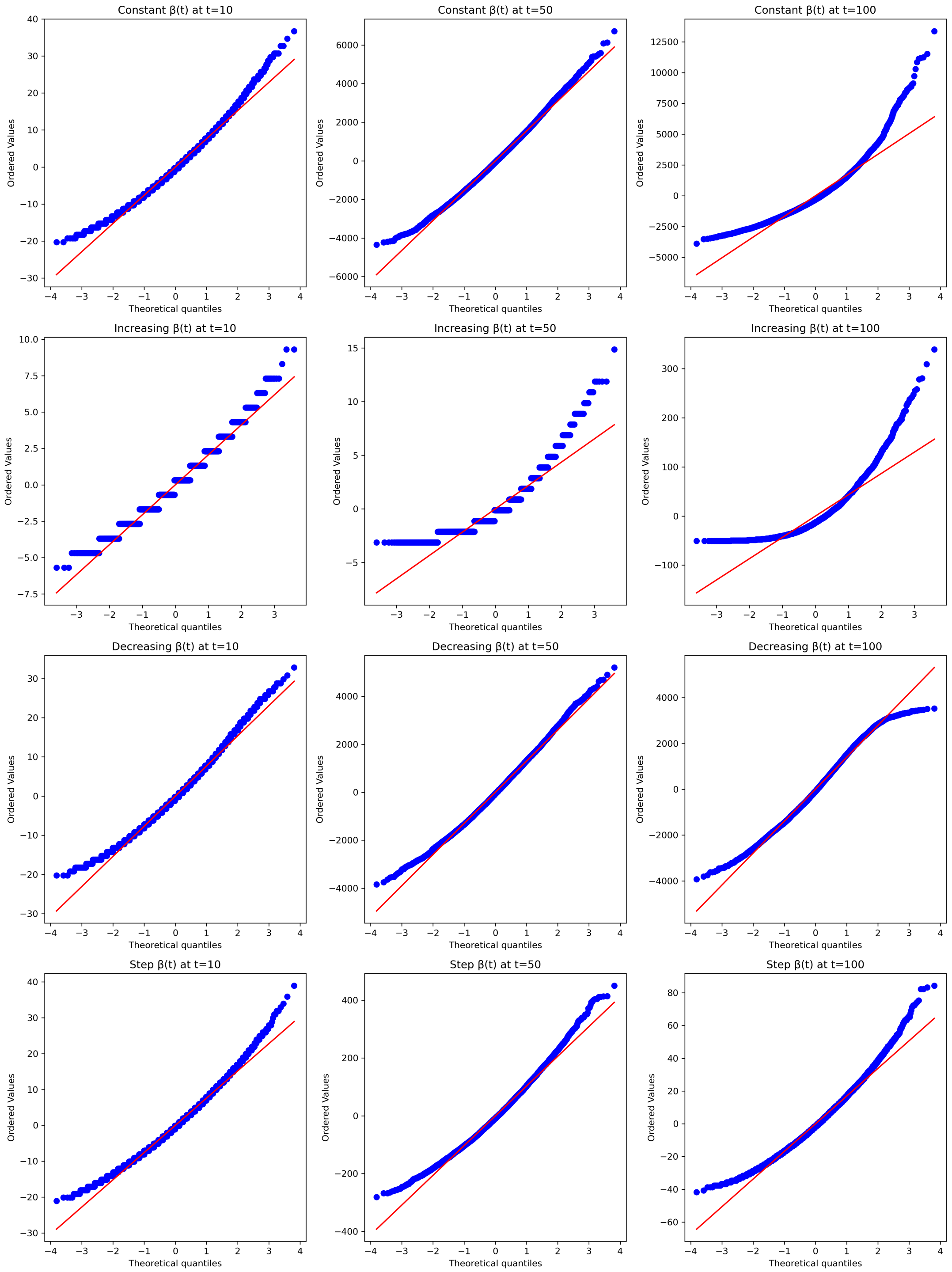

Fig S4A. Q–Q Plots for each of the four modelling scenarios (constant $\beta(t)$, increasing $\beta(t)$, decreasing $\beta(t)$ and a step-decrease in $\beta(t)$) at three different time points.
