# Supplementary material for: Identifying COVID-19 peaks using early warning signals
Source: PLoS Comput Biol. 2025 Sep 24;21(9):e1013524. doi: 10.1371/journal.pcbi.1013524 (PMC12483279; doi:10.1371/journal.pcbi.1013524)
Supplement: S5 Fig — For all figures: reported cases, effective reproduction number, variance between the mean-detrended simulations and time-of-detection distribution for the ten thousand simulations run for each of the four modelling scenarios (constant β(t), increasing β(t), decreasing β(t) and a step-decrease in β(t)). S5A: 80% reporting probability and dispersion of 10. S5B: 60% reporting probability and dispersion of 1. S5C: 60% reporting probability and dispersion of 10. (PDF) [file pcbi.1013524.s005.pdf]

Sensitivity analysis for case reporting distribution

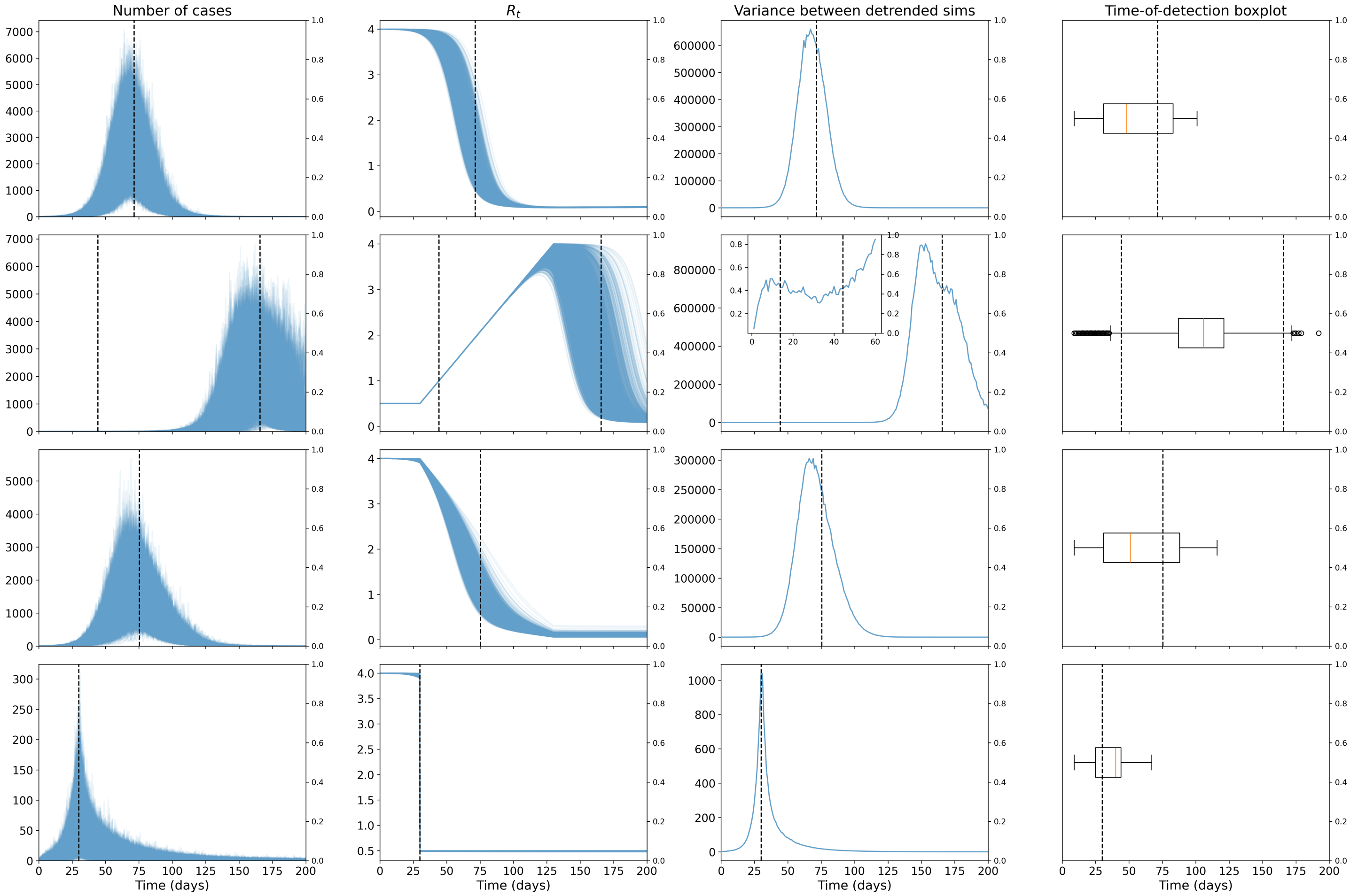

Fig. S5A. Reported cases, effective reproduction number, variance between the mean-detrended simulations and time-of-detection distribution for the ten thousand simulations run for each of the four modelling scenarios (constant $\beta(t)$, increasing $\beta(t)$, decreasing $\beta(t)$ and a step-decrease in $\beta(t)$) for an Alpha-like pathogen with an 80% reporting probability and dispersion of 10.

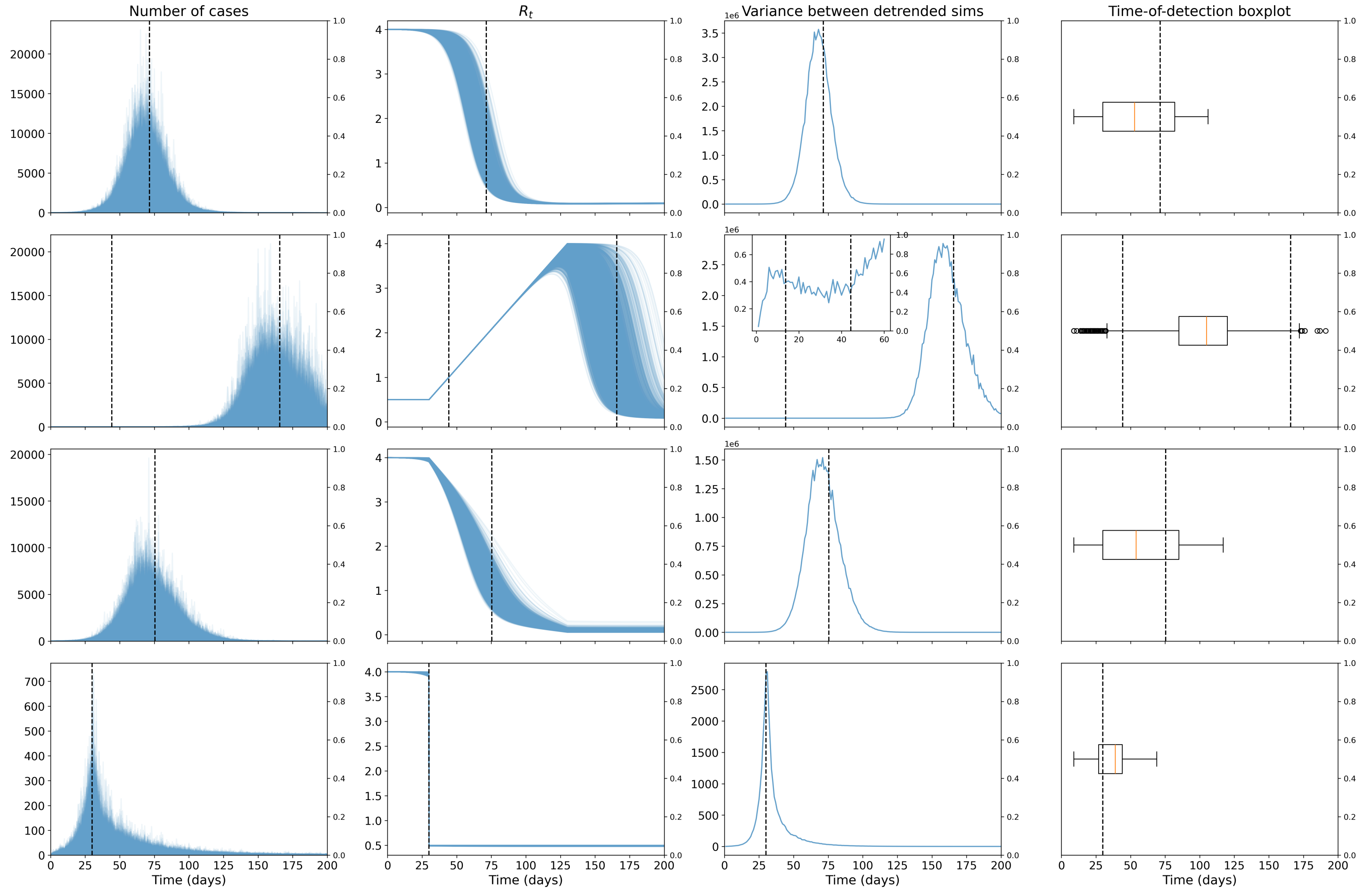

Fig. S5B. Reported cases, effective reproduction number, variance between the mean-detrended simulations and time-of-detection distribution for the ten thousand simulations run for each of the four modelling scenarios (constant $\beta(t)$, increasing $\beta(t)$, decreasing $\beta(t)$ and a step-decrease in $\beta(t)$) for an Alpha-like pathogen with a 60% reporting probability and dispersion of 1.

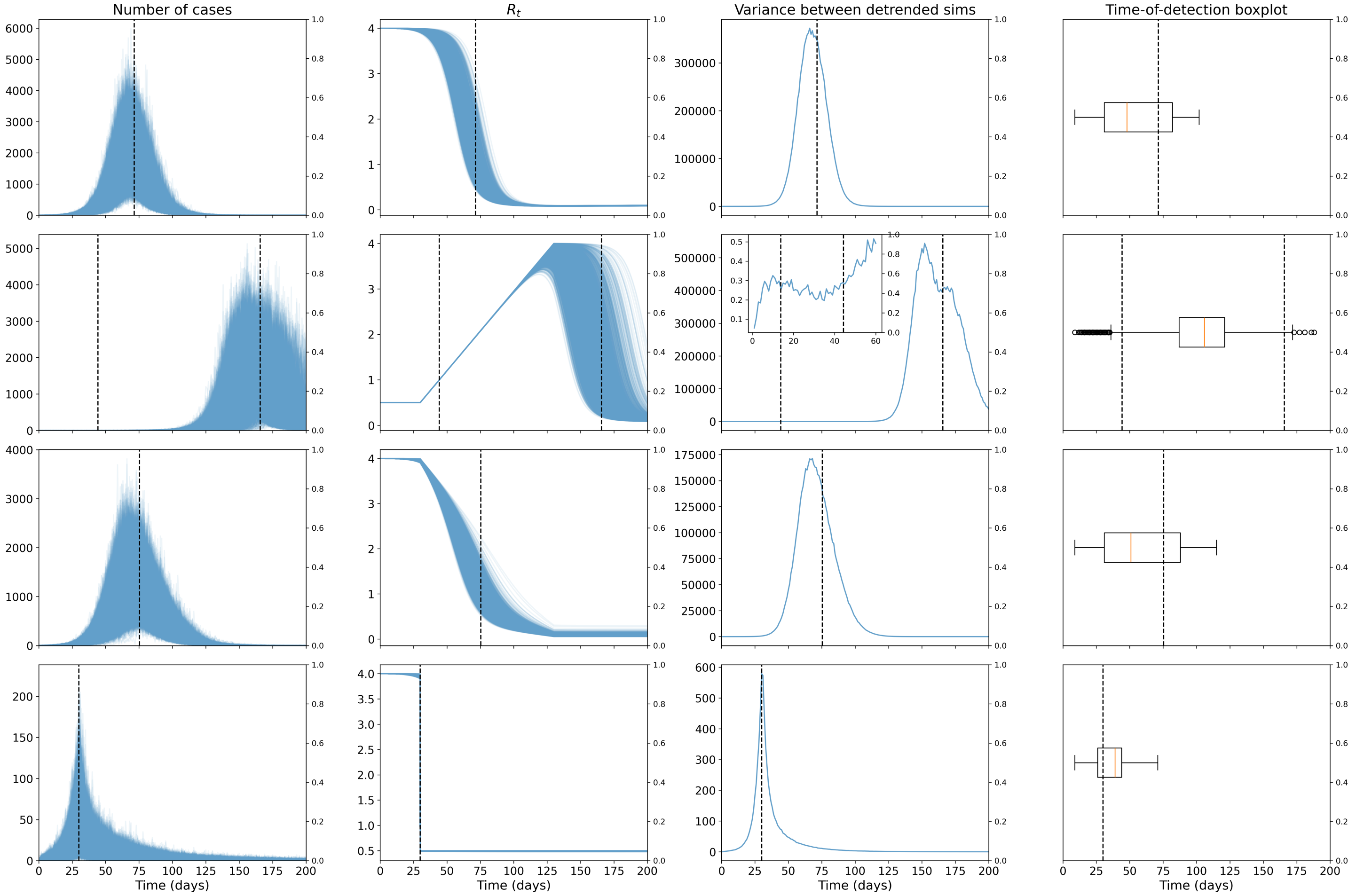

Fig. S5C. Reported cases, effective reproduction number, variance between the mean-detrended simulations and time-of-detection distribution for the ten thousand simulations run for each of the four modelling scenarios (constant $\beta(t)$, increasing $\beta(t)$, decreasing $\beta(t)$ and a step-decrease in $\beta(t)$) for an Alpha-like pathogen with a 60% reporting probability and dispersion of 10.
