# Supplementary material for: Identifying COVID-19 peaks using early warning signals
Source: PLoS Comput Biol. 2025 Sep 24;21(9):e1013524. doi: 10.1371/journal.pcbi.1013524 (PMC12483279; doi:10.1371/journal.pcbi.1013524)
Supplement: S6 Fig — Distribution shown is the proportion of total sequences represented by each (named) variant. (PDF) [file pcbi.1013524.s006.pdf]

Variants Timeline

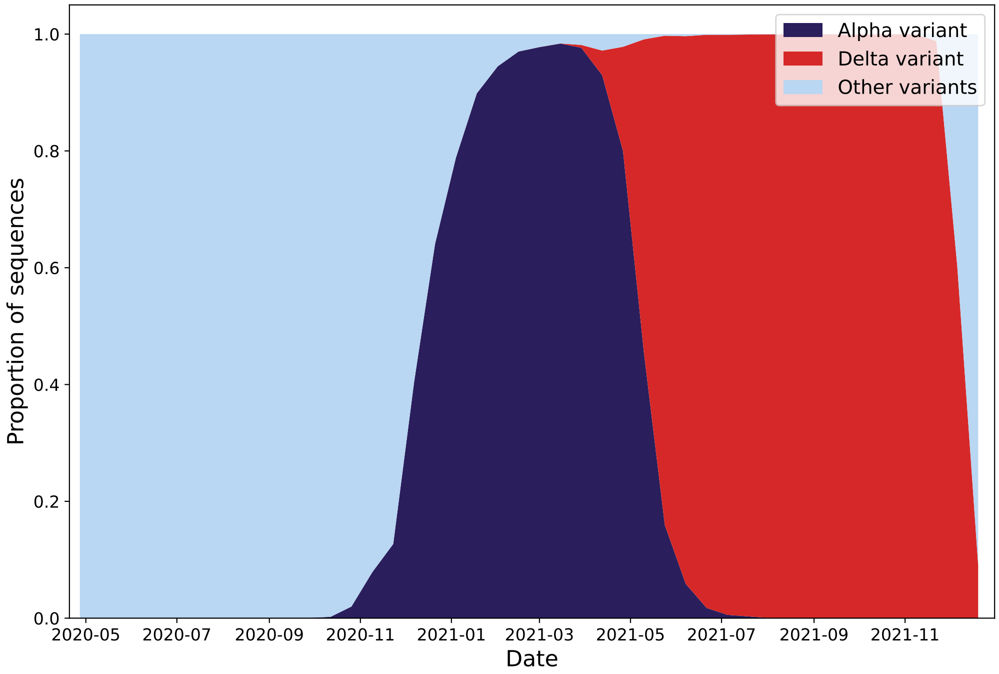

Fig. S6A. Timeline of the sequenced variants of COVID-19 in the UK between June 2020 and December 2021. Distribution shown is the proportion of total sequences represented by each (named) variant.
