# Supplementary material for: Identifying COVID-19 peaks using early warning signals
Source: PLoS Comput Biol. 2025 Sep 24;21(9):e1013524. doi: 10.1371/journal.pcbi.1013524 (PMC12483279; doi:10.1371/journal.pcbi.1013524)
Supplement: S7 Fig — All figures plot the time series of new COVID-19 cases and selected time series statistics for a sample of LTLAs between June 2020 and December 2021. S7A: Normalised statistics with a 7- day and 14- day window. S7B: Statistics with a 10- day and 30- day window. (PDF) [file pcbi.1013524.s007.pdf]

Sensitivity analysis of time series statistics to window size

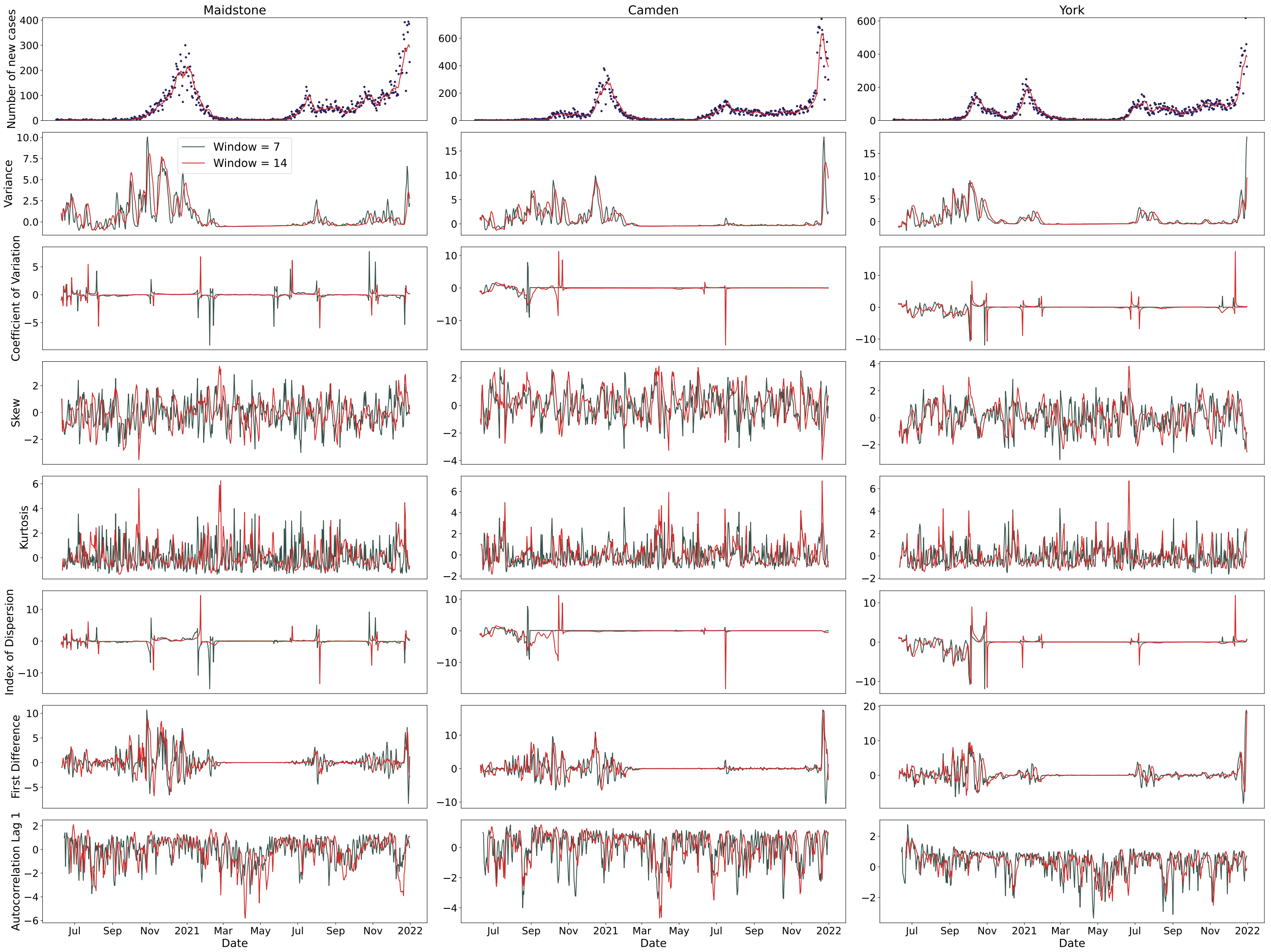

Fig. S7A. Time series of new COVID-19 cases and selected normalised time series statistics for a sample of LTLAs between June 2020 and December 2021. Statistics are calculated using a rolling 7- or 14-day window.

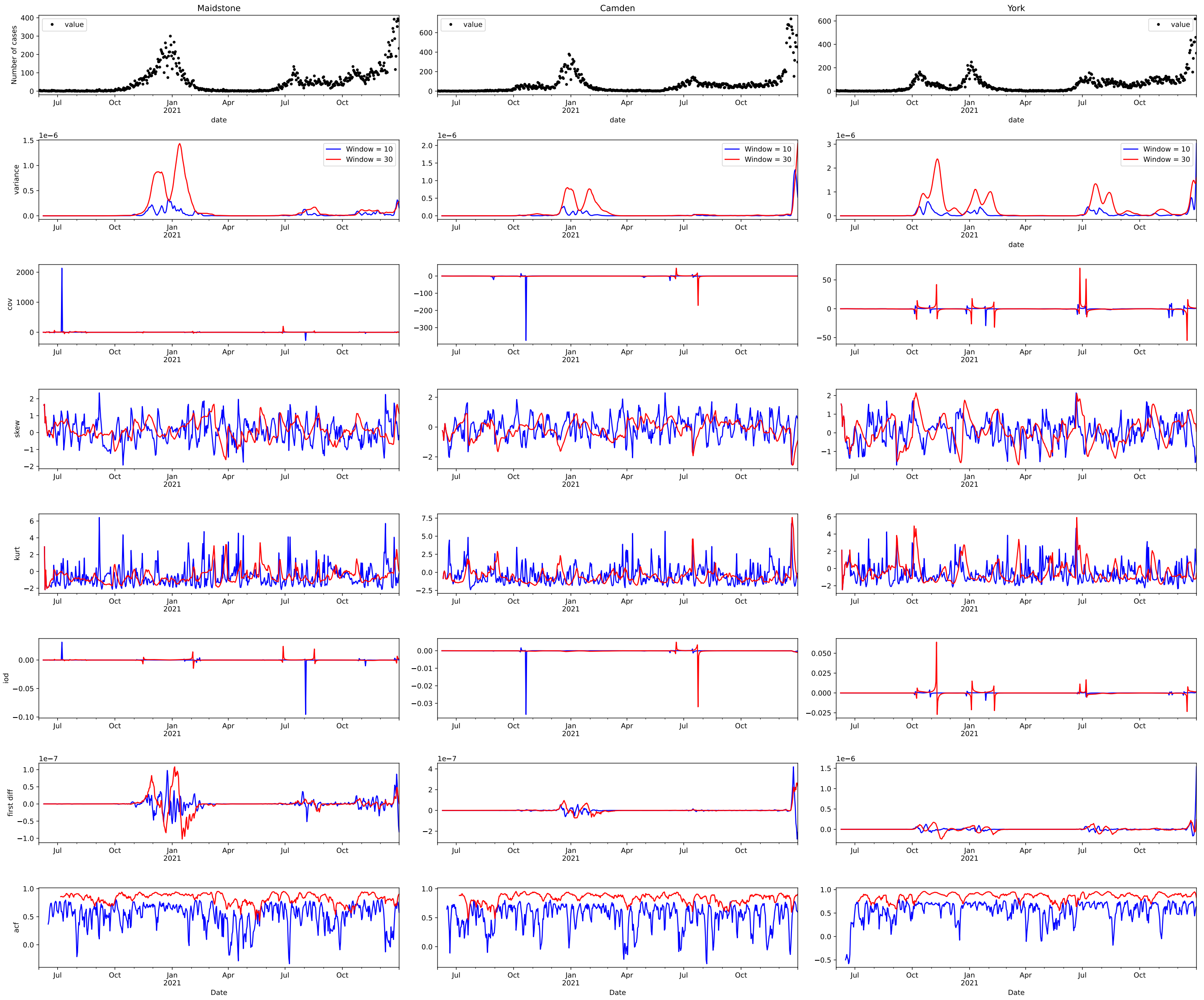

Fig. S7B. Time series of new COVID-19 cases and selected time series statistics for a sample of LTLAs between June 2020 and December 2021. Signals were calculated using a rolling 10- or 30-day window.
